# Three-dimensional human placenta-like bud synthesized from induced pluripotent stem cells

**DOI:** 10.1038/s41598-021-93766-9

**Published:** 2021-07-08

**Authors:** Mai Sato, Asako Inohaya, Eriko Yasuda, Haruta Mogami, Yoshitsugu Chigusa, Kaoru Kawasaki, Yosuke Kawamura, Yusuke Ueda, Hiroshi Takai, Masaki Mandai, Eiji Kondoh

**Affiliations:** Department of Gynecology and Obstetrics, Kyoto University Graduate School of Medicine, 54 Shogoin Kawahara-cho, Sakyo, Kyoto 606-8507 Japan

**Keywords:** Pluripotency, Stem cells

## Abstract

Placental dysfunction is related to the pathogenesis of preeclampsia and fetal growth restriction, but there is no effective treatment for it. Recently, various functional three-dimensional organs have been generated from human induced-pluripotent cells (iPSCs), and the transplantation of these iPSCs-derived organs has alleviated liver failure or diabetes mellitus in mouse models. Here we successfully generated a three-dimensional placental organ bud from human iPSCs. The iPSCs differentiated into various lineages of trophoblasts such as cytotrophoblast-like, syncytiotrophoblast-like, and extravillous trophoblast-like cells, forming organized layers in the bud. Placental buds were transplanted to the murine uterus, where 22% of the buds were successfully engrafted. These iPSC-derived placental organ buds could serve as a new model for the study of placental function and pathology.

## Introduction

The placenta is a multifunctional organ that supports the developing fetus. Improper placental development in the early stages of pregnancy can result in fetal growth restriction, preeclampsia, and miscarriage^1,2^. Surprisingly, there are few in-vitro experimental models of the early stages of human placental development, although these would improve our understanding of the physiological aspects of placentation and our ability to develop novel treatments for placental dysfunctions.

Trophoblasts are specialized cells of the placenta that originate from the trophectoderm, the outer layer of the blastocyst^3^. The placenta is composed of three types of trophoblasts: cytotrophoblasts (CTBs), syncytiotrophoblasts (STBs) and extravillous trophoblasts (EVTs). Proliferative CTBs can differentiate into either STBs or EVTs. STBs form a multinuclear layer in the outer surfaces of the placental villi and are responsible for gas/nutrient exchange and hormone production. EVTs invade the lumina of the spiral arteries as well as the decidua and the myometrium, where they contribute to anchoring the placenta and directing a sufficient supply of maternal blood into it. The differentiation of trophoblasts can be observed through changes in the expression patterns of specific gene markers. Trophoblast ectoderm (TE) is represented by caudal type homeobox 2 (CDX-2)/(*CDX2*). Tumor protein p63 (TP63)/*TP63* is a proliferative CTB marker and cytokeratin 7 (CK7)/*keratin 7* (*KRT7*) is a pan-trophoblast or CTB marker. The STB markers are human chorionic gonadotropin (hCG)/*chorionic gonadotropin subunit beta 3* (*CGB3*) and syncytin-1/*endogenous retrovirus group W member 1* (*ERVW1*). The EVT marker is human leukocyte antigen G (HLA-G)/(*HLAG*), and the cell column trophoblast marker is peroxisome proliferator-activated receptor gamma (PPAR-γ)/(*PPARG*).

Three-dimensional organ buds, including liver, kidney, and pancreas buds, have been generated in vitro by culturing tissue-specific progenitors from induced pluripotent stem cells (iPSCs) with human umbilical vein endothelial cells (HUVECs) and human mesenchymal stem cells (MSCs)^4–6^. Elsewhere, trophoblast-like cells have been created from human embryonic stem cells by adding bone morphogenetic protein 4 (BMP4)^7^. Here, we attempted to generate three-dimensional human placenta-like organ buds from iPSCs with the aid of endothelial and mesenchymal cells.

## Results

### Differentiation of iPS cells into trophoblast lineage by BMP4 treatment

**Figure 1:**
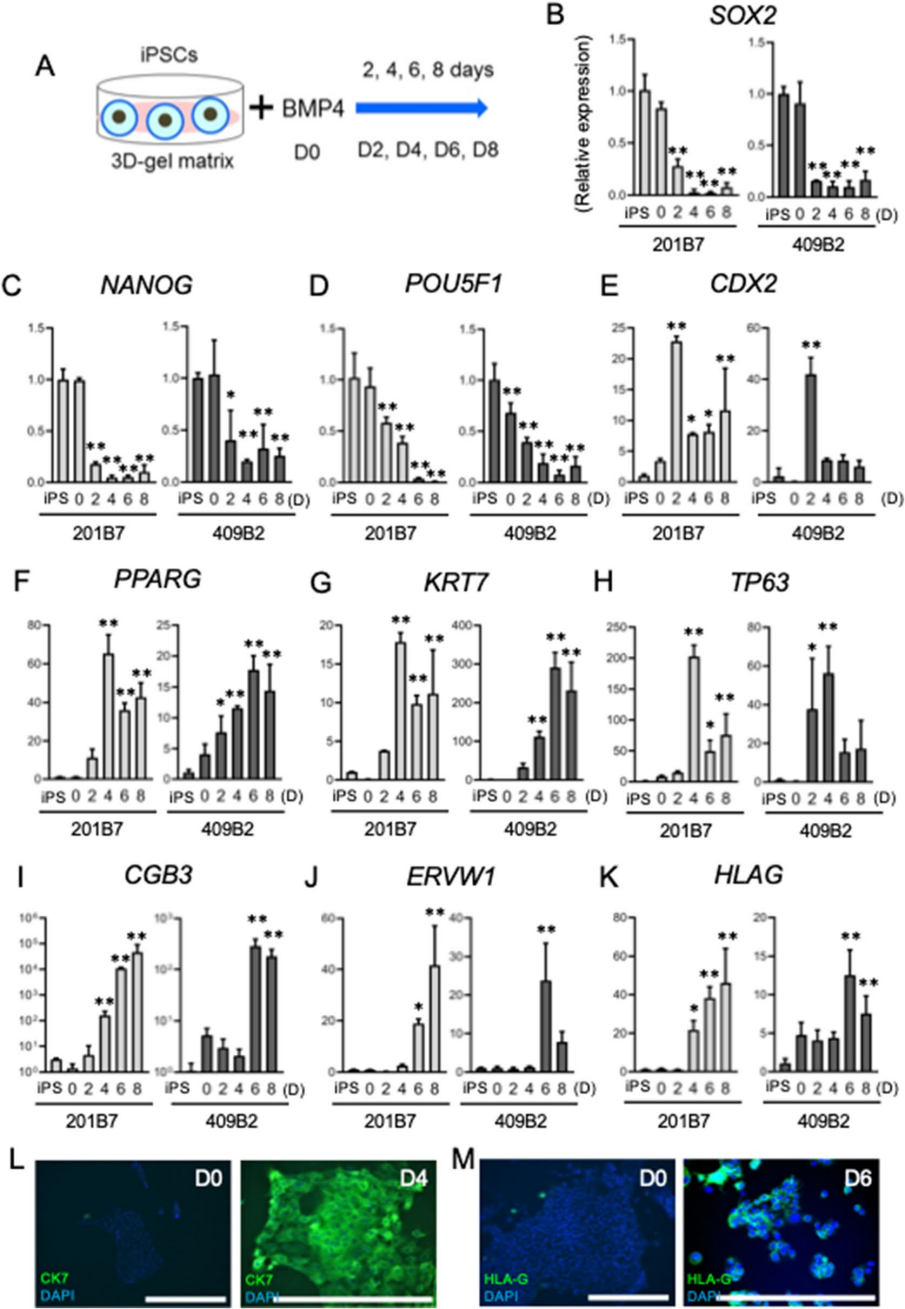
Trophoblastic differentiation of iPSCs by means of BMP4 treatment. (**A**) Our scheme for inducing trophoblast-like cells from iPSCs. iPSCs were treated with BMP4 for 2, 4, 6, and 8 days (D2, D4, D6, and D8, respectively). (**B**–**K**) mRNA expression of trophoblast markers in BMP4-treated iPSCs (201B7 and 409B2) from day 0 (D0) to day 8 (D8) of culture. Relative mRNA expression level of each gene was compared to the level of iPSCs, and statistical significance was analyzed by ANOVA. Error bars represent SD. n = 3 in each group. *P < 0.05, **P < 0.01. (L) Immunofluorescence of CK7 (green, upper panel) and HLAG (green, lower panel) in BMP4-treated iPSCs (201B7) at day 0, 4, and 6 (D0, D4, and D6, respectively). Nucleus was stained by DAPI (blue). Bars, 300 μm.

First, iPSCs were treated with 100 ng/mL of BMP4 in a three-dimensional (3D) gel matrix and cultured for eight days (Fig. 1A). To observe the subsequent differentiation of two cell lines of iPSCs (201B7 and 409B2) into trophoblast-like cells, we analyzed representative markers of the trophoblast lineage as a function of time (Fig. 1B–H). mRNA expression levels of the pluripotent cell markers, sex determining region Y-box2 (SOX2)/(*SOX2*), Nanog homeobox (*NANOG*), and octamer-binding transcription factor 4 (OCT4)/*POU class 5 homeobox 1* (*POU5F1*) were high in iPSCs and day 0 cells (Fig. 1B–D), but gradually decreased from day 2, indicating differentiation into trophoblasts. In contrast, mRNA expression levels of caudal type homeobox 2 (CDX2), a TE marker, was low in iPSCs and day 0 cells but abruptly increased after two days of BMP4 treatment, suggesting that iPSCs had started to differentiate into TE-like cells by this time (Fig. 1E). Starting on day 4, however, this increase was reversed: CDX2 expression decreased to and then remained at a modest level, suggesting the further differentiation of TE-like cells into CTB- and STB-like cells (Fig. 1E).

The mRNA levels of *PPARG*, *KRT7*, and *TP63* were low in iPSCs and day 0 cells, but increased from day 2, reaching a peak at day 4 in 201B7 cells (Fig. 1F–H). On the other hand, in 409B2 cells, the mRNA levels of those genes peaked on day 6 (Fig. 1F–H). This suggests that on days 4–6 of BMP4 treatment, iPSCs differentiate into cell column trophoblast- and CTB-like cells, and that the rate of differentiation into CTBs is relatively slow in 409B2 compared to 201B7. Subsequently, the mRNA expression levels of *PPARG*, *KRT7*, and *TP63* decreased slightly (Fig. 1F–H), suggesting that these cells further differentiated into STB-like cells after day 4 of BMP4 treatment, although they retained some degree of CTB character.

The mRNA expression levels of the STB markers human chorionic gonadotropin (hCG)/ *chorionic gonadotropin subunit beta 3* (*CGB3*) and syncytin-1/*endogenous retrovirus group W member 1* (*ERVW1*) remained low from the iPSC stage through day 2–4, suggesting that differentiation into the STB phenotype did not occur until day 2–4 (Fig. 1I,J). From day 4 in 201B7 and day 6 in 409B2, however, *CGB3* and *ERVW1* mRNA increased (Fig. 1I,J), indicating that BMP-4-treated iPSCs were differentiating into STB-like cells. Similarly, *human leucocyte antigen-G* (*HLAG*) mRNA increased from day 4–6 of BMP4 treatment (Fig. 1K), implying that some portion of iPSCs were differentiating into EVT-like cells. Since CTB-like differentiation was relatively slower in 409B2 than in 201B7 (approximately 2 days behind), the rate of differentiation into STB- and EVT-like cells were concomitantly slower in 409B2 compared to 201B7.

Next, we compared the expression levels of cytokeratin-7 (CK7) and HLA-G between day 0 and 4 of BMP4 treatment by immunofluorescence in 201B7 iPSCs. CK7 was not detected before BMP4 treatment (day 0) but was highly expressed in the cytoplasm on day 4 (Fig. 1L). Similarly, HLA-G was not expressed on day 0 but was observed around the nucleus on day 6 (Fig. 1M). These changes in CK7 and HLA-G protein expression were compatible with those in their mRNA expression (Fig. 1G,K). Collectively, these results demonstrate that iPSCs were successfully differentiated into trophoblast-like cells by BMP4 treatment, and this trophoblast differentiation was universally observed in different iPS cell lines.

### Generation of three-dimensional placental organ buds from iPSCs

**Figure 2:**
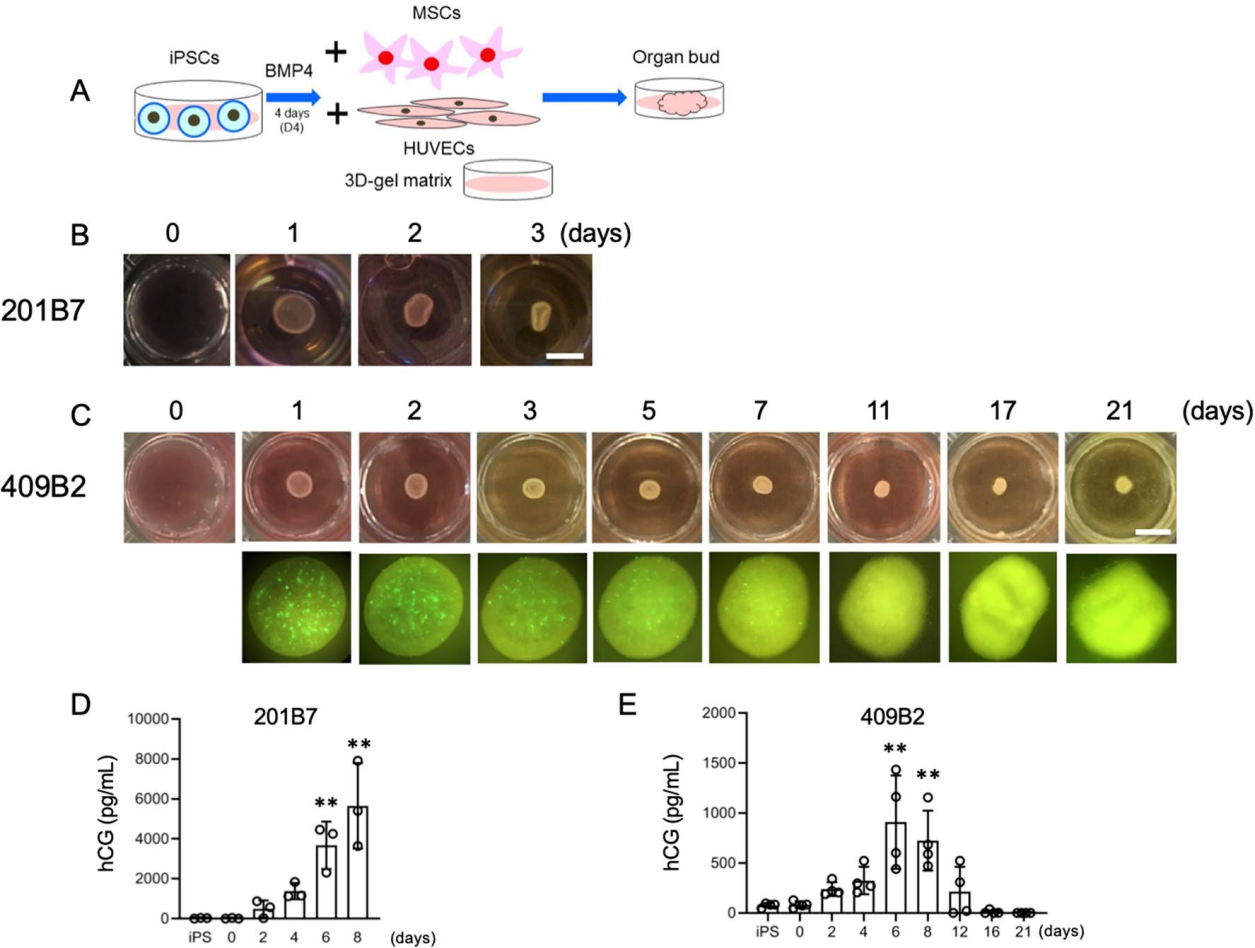
Placental organ buds synthesized from iPSCs. (**A**) Our scheme for synthesizing placental organ buds from iPSCs. (**B**) Representative images of three-dimensional placental buds after co-culture with HUVECs and MSCs for 0–3 days. 201B7 iPSCs treated with BMP4 for 4 days (D4) were utilized. Bars, 5 mm. (**C**) Representative images of placental buds after co-culture with HUVECs and MSCs for 0–21 days. 409B2 iPSCs treated with BMP4 for 4 days (D4) were utilized. Macroscopic views (upper panels) and the views under fluorescence microscope (lower panels). Note that 409B2 iPSCs contain GFP, so the iPSCs were identified by green fluorescence. Bars, 5 mm. (**D**) hCG levels in the medium of placental organ buds from 0 to 8 days. 201B7 iPSCs treated with BMP4 for 4 days (D4) were utilized. iPS represents the cells before BMP4 treatment. hCG levels were statistically analyzed by ANOVA, and the hCG level on each day was compared to that of iPSCs in post-hoc test. n = 3 in each group. (**E**) hCG levels in the medium of placental organ buds from 0 to 21 days. 409B2 iPSCs treated with BMP4 for 4 days (D4) were utilized. iPS represents the cells before BMP4 treatment. hCG levels were statistically analyzed by ANOVA, and the hCG level on each day was compared to that of iPSCs in post-hoc test. n = 3 in each group. **, *P* < 0.01.

Next, we attempted to generate a placental organ bud from iPSCs. We began this experiment with iPSCs that had been stimulated with BMP4 for four days. Trophoblast-like cells treated with BMP4 were mixed with human mesenchymal stem cells (MSCs) from the amnion and human umbilical vein endothelial cells (HUVECs) from the umbilical cord in a 3D-gel matrix (Fig. 2A). One day after mixture, they started to self-organize into three-dimensional cells in both 201B7 and 409B2 iPSCs (Fig. 2B,C). Self-organization did not occur in the absence of HUVEC or MSCs (Supplementary Fig. 1). We were also able to generate placental buds from trophoblast-like cells that had been treated with BMP4 for either 2 or 6 days, but not for 8 days (Supplementary Fig. 2). To characterize the STB-like differentiation and viability in these placental organ buds, we assayed hCG levels in the conditioned medium as a function of time. The hCG levels in the culture medium of placental buds derived from 201B7 iPSCs gradually increased from day 2 to 8 after seeding (Fig. 2D). The culture period of organ buds created using 409B2 iPSCs was extended to 21 days (Fig. 2C). The size of the buds remained apparently unchanged until day 7–8, but gradually shrank from day 11–12. Similar to the placental buds derived from 201B7 iPSCs, the concentration of hCG in the culture medium also gradually increased and reached a peak on days 6–8. However, it then began to decrease, reaching almost zero on days 16 and 21 (Fig. 2E). In addition, the number of GFP-labeled 409B2 iPSCs gradually decreased in the placental buds, becoming almost nonexistent on days 17 and 21. These data suggest that the placental organ buds remain functional for at least one week, but begin to weaken beyond about two weeks and may not be viable by three weeks.

**Figure 3:**
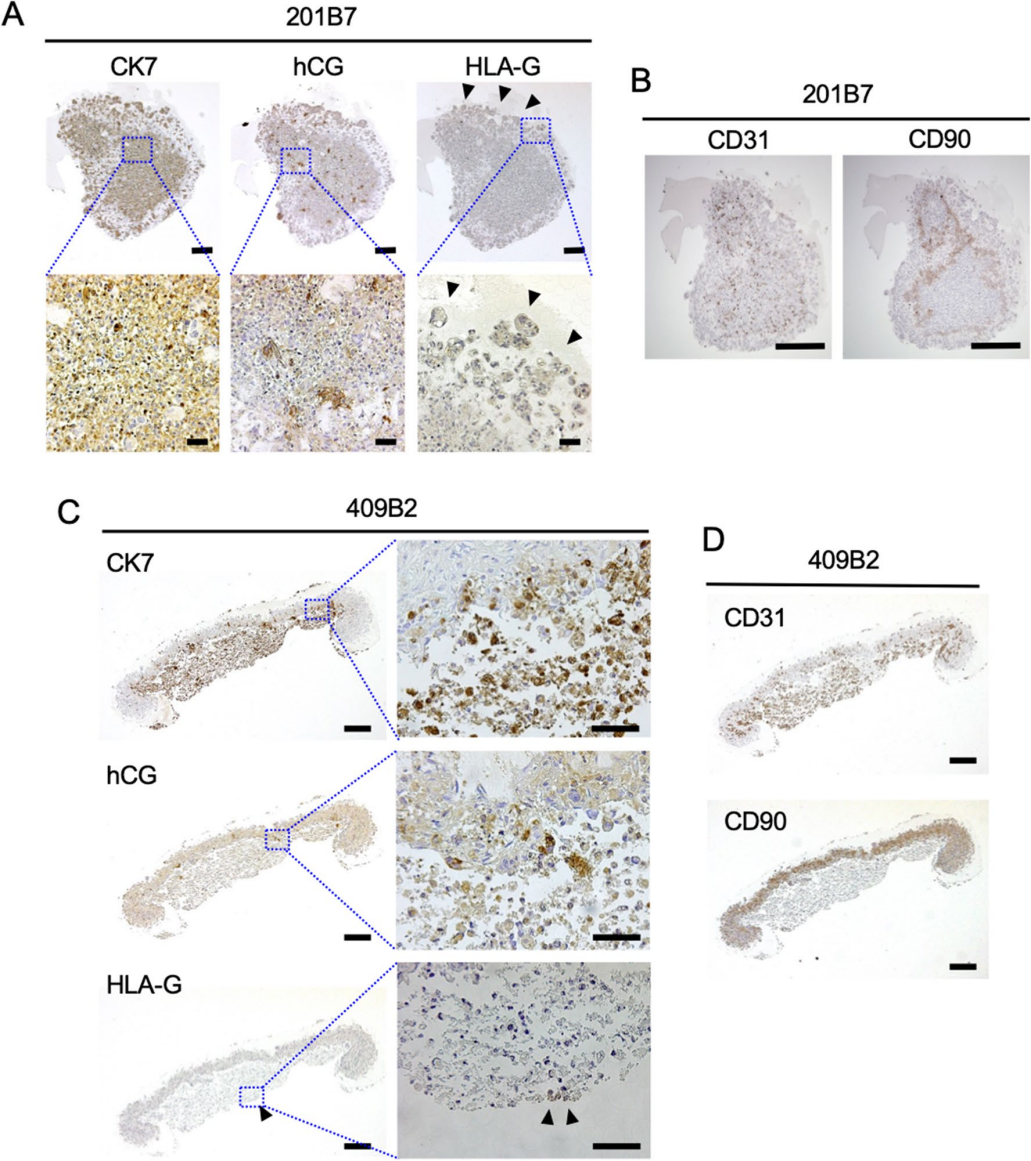
Expressions of trophoblast makers in the placental organ buds. (**A**, **C**) Immunohistochemistry of CK7, hCG, and HLAG in a placental organ bud from 201B7 iPSCs (**A**) or 409B2 iPSCs (**C**). Arrows indicate staining for HLA-G. Bars, 250 μm in lower magnification and 50 μm in higher magnification. (**B**, **D**) Immunohistochemistry of CD31 and CD90 in an organ bud from 201B7 iPSCs (**B**) and 409B2 iPSCs (**D**). Bars, 500 μm.

Immunohistochemical analysis of a placental bud obtained from 201B7 iPSCs revealed that CK7 was ubiquitously expressed in the organ bud (Fig. 3A). hCG was expressed inside the organ bud, where it formed island-like aggregates (Fig. 3A). In contrast to hCG, HLA-G staining, although less intense than CK7 and hCG, was observed at the periphery compared to the interior of the bud (Fig. 3A), implying that these trophoblast-like cells were forming layers dependent on their character. CD31 (the marker for HUVECs)- and CD90 (the marker for MSCs)-positive cells were located in different places in the bud (Fig. 3B). The localization of differentiated iPSCs-derived trophoblast-like cells, HUVECs, and MSCs was similar in the placental bud generated using 409B2 iPSCs (Fig. 3C,D). This indicates that the added HUVECs and MSCs maintain their original properties, but during organ bud differentiation, both cells seem to migrate to different locations. Collectively, our results demonstrated that induced-trophoblast cells from iPSCs were able to form three-dimensional structures with the assistance of MSCs and HUVECs. In addition, differentiated trophoblast-like cells formed layers within the bud. Syncytium-like cells as identified by hCG were localized inside the bud, while EVT-like cells as identified by HLA-G formed the outer layer of the bud.

### Transplantation of placental bud to the NOD/SCID mouse uterus

**Figure 4:**
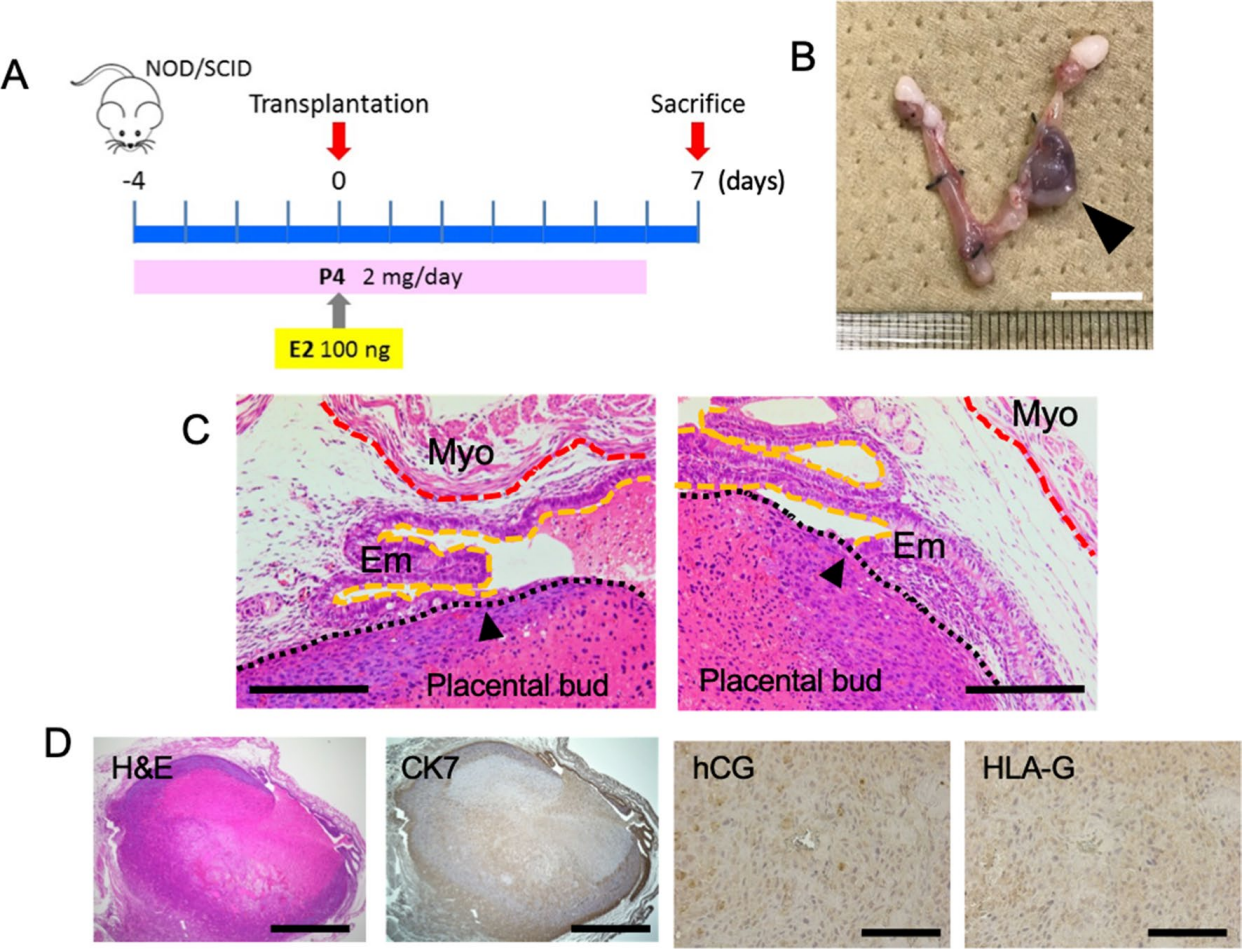
Transplantation of placental buds into NOD/SCID mice. (**A**) A schematic diagram of placental bud transplantation. E2, estradiol; P4, progesterone. (**B**) Macroscopic image of the engrafted placental bud (case 1). Bar, 1 cm. (**C**) H&E staining of engrafted placental organ bud (case 1). A black arrow head indicates the border between bud and endometrium. Myo: myometrium, Em: endometrium. Bars, 200 µm. (**D**) H&E staining and immunohistochemistry of CK7, hCG, and HLAG of the transplanted organ bud (case 1). Bars, 1 mm (H&E and CK7), 100 µm (hCG and HLA-G).

Finally, we tested the orthotopic transplantation of these placental organ buds into immunodeficient mice. To induce a pseudo-pregnant condition, a total of nine NOD/SCID mice were pretreated with progesterone starting four days before transplantation (Fig. 4A). On the day of transplantation, 100 ng of estradiol was subcutaneously injected. After laparotomy, placental buds were transplanted into the uterine lumen using a syringe with an 18 gauge needle. Hormonal supplementation was administered for seven days and engraftment was judged on the seventh day after transplantation (Fig. 4A). We observed that placental buds had successfully engrafted and survived in two of the nine injected mice (22%). These two engrafted organ buds were then histologically examined.

In case 1, the placental organ bud (Fig. 4B) was attached to the endometrium (Fig. 4C). The engrafted organ bud expressed CK7, hCG, and HLA-G (Fig. 4D).

**Figure 5:**
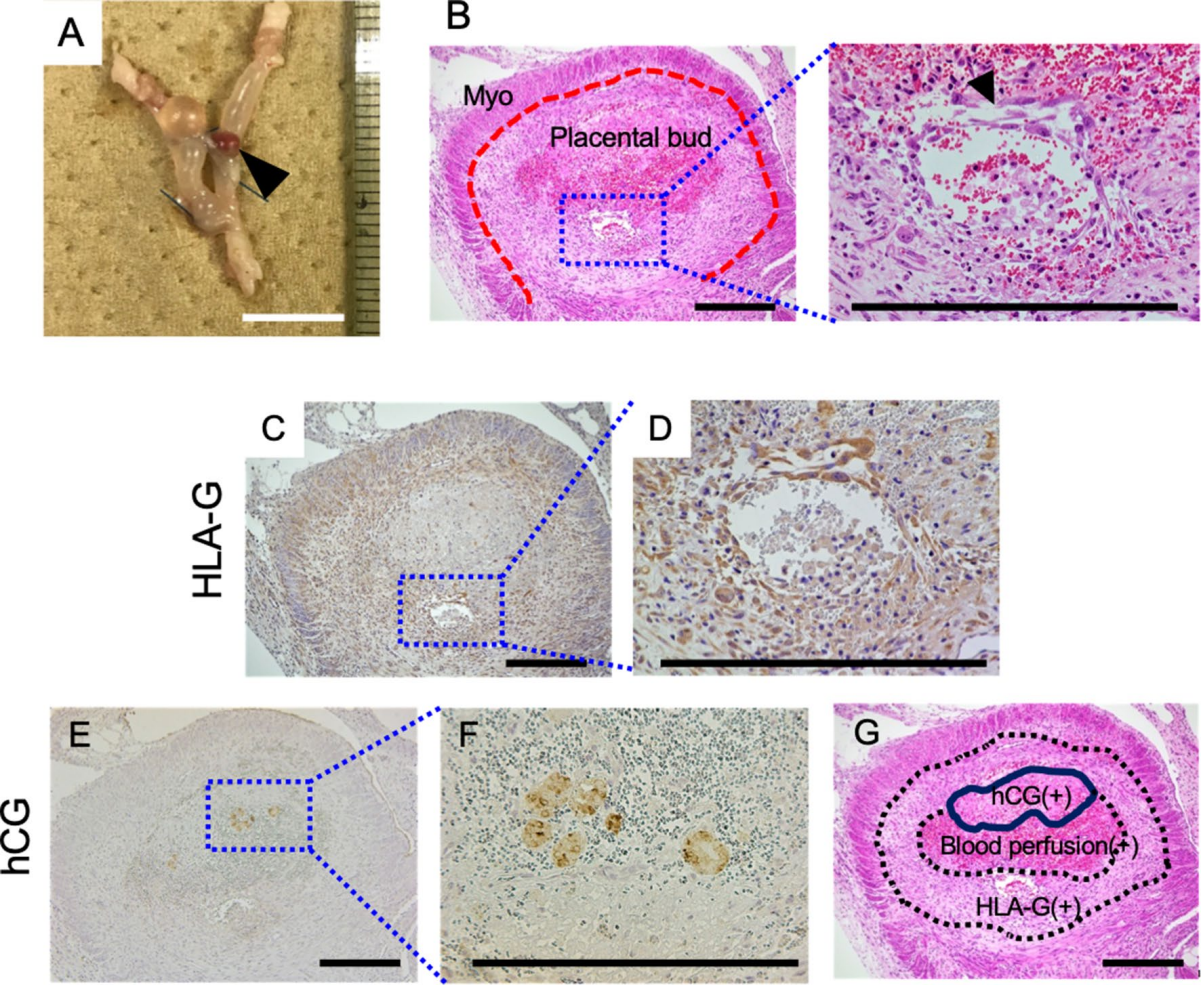
Trophoblast layers forming in the transplanted placental organ bud. Histological examination of case 2. (**A**) Macroscopic image of the transplanted organ bud in the NOD/SCID mouse uterus (case 2). A black arrow head indicates the bud. Bar, 1 mm. (**B**) H&E staining of case 2. Lower (left) and higher magnification (right). Black arrow head indicates the vascular-like structure inside the organ bud. (**C**, **D**) Immunohistochemistry of HLA-G. (**C**) Lower magnification (middle), and (**D**) higher magnification of the vasculature-like structure. (**E**, **F**) Immunohistochemistry of hCG. (**E**) Lower and (**F**) higher magnification. (**G**) Scheme of the trophoblast layers and blood-perfused area in the organ bud. All bars, 300 µm.

In case 2, the placental organ bud (Fig. 5A) was engrafted inside the uterus (Fig. 5B). Interestingly, a blood vessel-like structure was observed in the center of the bud (Fig. 5B). HLA-G-positive EVT-like trophoblast cells were observed beneath the attachment site, forming an outer layer of the bud (Fig. 5C). In addition, HLA-G was also expressed in cells that construct blood vessel-like tissues in the buds of organs (Fig. 5D). Next to this EVT-like layer, hCG-positive cells formed islets inside the bud (Fig. 5E,F). These inner hCG-positive islets were clearly separated from the outer HLA-G-positive EVT-like layer (Fig. 5G). The EVT layer and the hCG islets surrounded a blood perfusion area that was rich in red blood cells (Fig. 5G). Again, the attached placental organ bud was able to differentiate properly in vivo such that EVT-like cells resided outside the bud and hCG-positive STB-like cells were localized next to the EVT-like cells. This suggests that a transplanted organ bud can sense its surrounding environment, and that signals from the environment might direct bud migration and differentiation in the proper direction.

## Discussion

Using human iPSCs and immunodeficient mice, we successfully demonstrated that (1) trophoblast-like cells can be induced with BMP4 treatment, (2) three-dimensional placenta-like organ buds can be induced from iPSCs with the aid of MSCs and HUVECs in a 3D-gel matrix, and (3) synthesized placental organ buds can be transplanted to the uteruses of immunodeficient mice, whereupon trophoblast-like cells can migrate and differentiate in the proper direction within the organ bud.

### (1) BMP4 induces trophoblast differentiation from iPSCs

In 2002, Xu et al. succeeded in differentiating human embryonic stem cells (ES cells) into trophoblasts by adding BMP4^7^. In their experiment, trophoblast-like morphological changes were observed on day 2 of treatment with 100 ng/mL BMP4. We followed their methods in our iPSC experiment and observed that our iPSCs lost their pluripotency (as evidenced by the decrease in *SOX2*, *NANOG*, and *POU5F1* mRNA) and gained trophoblast-like features (as evidenced by the increases in *CDX2*, *PPARG*, *KRT7*, *TP63*, and *CGB3* mRNA) from day 2. The trophoblast-like differentiation pattern of iPSCs induced by BMP4 is very similar to that of ES cells by Xu’s. The methodological difference between the two is the cell source: Xu used ES cells, whereas we used iPSCs. ES cells are derived from human blastocysts, which poses ethical concerns. The advantage of using iPSCs is that iPSCs can be produced from somatic cells derived from skin and blood, and those somatic cells can be harvested relatively easily and with fewer ethical issues. Moreover, since iPSCs can be made from the patient’s own somatic cells, there is less risk of rejection in the clinical application of cell and organ transplantation. In 2016, Horii et al. showed that human pluripotent stem cells can be differentiated into CTB-like cells by treatment with low-dose BMP4 (10 ng/mL)^8^. Further, these cells can be differentiated into STBs and EVT-like cells in the presence of feeder-conditioned medium containing 10 ng/mL of BMP4^8^. Thus, BMP4 seems to be a master regulator in the early stages of human trophoblast differentiation^9^.

In human embryos, the pluripotency-associated transcription factor OCT4/*POU5F1* is initially expressed in the eight-cell stage at three days post-fertilization (dpf)^10^. OCT4/*POU5F1* expression was high in both the inner cell mass and the TE cells at 5 dpf, but was downregulated in the TE cells by 6 dpf^10^. In our iPSCs, downregulation of *POU5F1* mRNA started around day 2 of BMP4 treatment, which suggests that our iPSCs started to differentiate into TE cells around this stage. It is worth noting that, in mouse ES cells, forced repression of Oct3/4 induces differentiation toward the TE lineage, whereas overexpression of Oct3/4 induces differentiation into extraembryonic endoderm^11^. This suggests that the suppression of OCT4/*POU5F1* is necessarily for stem cells to differentiate toward the TE lineage.

The transcription factor CDX2 also appears to regulate cell differentiation toward TE in early embryos. In human embryos, CDX2 is not expressed at the eight-cell or morula stage at 3–4 dpf; rather, it is first detectable in TE at 5 dpf^10^. Interestingly, CDX2 expression overlaps with that of OCT4 in TE cells at 5 dpf^10^. In mouse embryos, CDX2 is required for the maintenance of TE and trophoblast stem cells^12^. Both downregulation of Oct3/4 and upregulation of Cdx2 can trigger the differentiation of mouse ES cells toward TE^12^. Similarly, *Cdx2* was required for the development of a functional TE lineage in a mouse *Cdx2*^-/-^ blastocyst experiment^13^. *Cdx2* is essential for the segregation of the ICM and TE lineages at the blastocyst stage by ensuring the repression of *Oct4* and *Nanog* in the TE lineage^13^. Thus, a well-coordinated transition from OCT4 to CDX2 seems to be critical for the differentiation of iPSCs into TE cells.

TP63, which is expressed in proliferative CTBs, is known to play an important role in maintaining of the stem cell sate of CTBs^14^. BMP4-treated human ES cells show decreased expression of TP63 during differentiation from CTBs to STBs, and forced down-regulation of TP63 inhibits differentiation of human ES cells into functional trophoblasts^15^. In the present experiments, it was confirmed that *TP63* was transiently increased in BMP4-treated iPSCs, and the expression changes of other trophoblast-related markers showed that the addition of BMP4 induced iPSCs to differentiate into CTB-like cells and then into STB-like and EVT-like cells.

### (2) Placental organ bud

Takebe et al. succeeded in producing a liver organ bud from iPSCs^6^, with MSCs and HUVECs helping to induce a 3D structure. These stromal cell populations are necessary not only for forming a 3D structure but also for inducing self-condensation^5^. Takebe et al. also used their self-condensation method to produce diverse organ buds such as a pancreatic islet^5^. In our study, likewise, we were not able to produce a placental organ bud in the absence of MSCs. The presence of HUVECs and MSCs is also necessary for generating vascularized tissues; the addition of HUVECS, in particular, enables the rapid formation of a functional vasculature^5^. Takebe et al. also reported that HUVECs and MSCs release various growth factors and cytokines that enhance the survival and functionality of organ buds^5^. MSCs can differentiate into osteocytes, adipocytes, chondrocytes, and cardiomyocytes, and were initially expected to serve as "cell replacements^16^. Recently, however, the role demanded of MSCs has been changing to that of "paracrine providers"^16^. MSCs produce a variety of growth factors, including vascular endothelial growth factor, fibroblast growth factor-2, and M-, G-colony stimulating factors. MSCs also secrete cytokines such as IL-10 and TGF-β in a paracrine manner and have anti-inflammatory effects. Therefore, 3D placental organs with MSCs may be advantageous for the implantation of placental organ buds.

Trophoblast organoids were generated by Turco et al.^17^, who used enzymatically digested first-trimester placentas to prepare trophoblast cells. Their organoids then differentiated into both STBs and EVTs^17^. The difference between this study and ours is that Turco et al. used cells that had already differentiated into trophoblasts to make their placental organoids, whereas we started with undifferentiated iPSCs. The use of first trimester villi in making organoids raises the possibility that certain gene expression patterns and epigenetic changes that are unique to the woman whose placenta is used could be passed to the organoids, so that the characteristics of organoids from different women’s placental cells would be heterogeneous. The use of iPSCs precludes this heterogeneity problem. While the organoid auxiliary cells, HUVECs and MSCs, are obtained from different persons, the iPSCs themselves are homogeneous because they are made from a single person. This is the main benefit of using iPSCs: it makes experiments reproducible by making the placental organ buds comparably homogeneous. Liang et al. successfully differentiated human iPSCs to MSCs^18^, and these iPSCs-derived MSCs significantly ameliorated limb ischemia in mice compared to adult bone marrow-derived MSCs. Therefore, it remains to be investigated whether placental buds with HUVECs, MSCs, and trophoblast-like cells derived from a single individual's iPSCs will increase their efficacy and viability.

### (3) Transplantation of placental organ buds

Takebe et al. transplanted functional liver organ buds from iPSCs to the mesentery^6^. These transplantations improved the survival rates of mice with liver dysfunction. Their group also transplanted pancreatic islets from iPSCs to under the renal capsules in diabetic mice, which likewise improved survival by normalizing blood glucose levels and stimulating insulin secretion^5^. Using their methods, we orthotopically transplanted placental organ buds into the uteruses of immunodeficient mice. In our transplanted organoids, STB-like cells were localized in the inner areas, whereas EVT-like cells were localized in the outer layers of the organ buds, which contacted the endometrium of the host uterus. The formation of organized trophoblast layers in the transplanted organ buds is very similar to the structure of early stages of human placenta^20^. This differentiation of SVT- and EVT-like cells into their proper directional orientation is an important finding in this transplantation study.

One limitation of this study is its lack of functional testing of the engrafted buds, which was not practical because the placenta has so many functions, ranging from nutrient and oxygen transport to hormone secretion and others. Further studies to analyze the long-term effects of bud transplantation, including regeneration of the dysfunctional placenta, tumorigenic potential, and improvement of FGR, are required to confirm the usefulness of this model.

### Summary

We have successfully generated three-dimensional placental buds from iPSCs and engrafted them into immunodeficient mice. Our placental organ buds may be a useful model for investigating the pathogenesis of and potential treatments for placental dysfunction.

## Materials and methods

### iPSC culture

Human iPS cell lines (201B7 and 409B2) were purchased from the RIKEN Cell Bank (#HPS0063 and #HPS0076, respectively). Mitomycin C-treated mouse embryonic fibroblast (MEF) feeder cells, derived from embryonic day 14.5 ICR mouse embryos, were cultured in Dulbecco's Modified Eagle Medium (#11960-044, Gibco) with 10% fetal bovine serum (#10270, Gibco), 2 mM L-glutamine (G7513, Sigma), and 1% penicillin/streptomycin (#15140122, Gibco). The iPSCs were grown on MEF feeder cells on gelatin-coated plates and maintained in Primate ES Cell Medium (RCHEMD001, ReproCELL) supplemented with 4 ng/ml recombinant human basic fibroblast growth factor (#064-04541, Wako) at 37 °C in humid air with 5% CO_2_. Undifferentiated colonies were passaged as small clumps using dissociation solution and pipettes, and split at a ratio of 1:3 every three days.

### Trophoblast-like differentiation of iPSCs

For trophoblast differentiation, we used the method of Xu et al. with slight modifications^7^. Briefly, differentiation was initiated by switching the medium to KnockOut DMEM/F12 (#12660012, Gibco) containing 2% bovine serum albumin (#A10008-01, Gibco), 2 mM L-glutamine (#G7513, Sigma), 10 µg/ml insulin-transferrin-selenium (#51500056, Gibco), 100 ng/ml heparin sulfate (#GAG-HS01, Iduron), and 0.1 mM non-essential amino acids solution (#11140050, Gibco). After two days, the cells were seeded into Geltrex (#A1413201, Gibco)-coated six-well plates in KnockOut DMEM/F12 containing 100 ng/ml recombinant human BMP4 (#314-BP-010, R&D Systems), 20% KnockOut Serum Replacement (#10828010, Gibco), 0.1 mM non-essential amino acids solution, and 0.1 mM β-mercaptoethanol (#21417-52, Nacalai Tesque). Incubated cells were collected on days 2, 4, 6, and 8.

### Isolation of human mesenchymal stem cells from amnion

Separation and isolation of MSCs were performed as previously described^21^. Human fetal membranes were obtained during caesarean deliveries, and the amnion was manually separated from the chorion beneath. Amnion tissues were then minced with scissors and digested with brightase-C/dispase 1 solution (brightase-C 40 μg/ml, #892431, Nippi, and dispase 1 200PU/ml, #386-02271, Wako) for 90 min at 37 °C with shaking. Digested tissues were filtered through a mesh strainer (Falcon #352360, Thermo Fisher Scientific) and centrifuged at 400 × g for 5 min at room temperature. The dissociated amnion cells were suspended in minimal essential medium (MEM) α (#12571063, Gibco) supplemented with 10% fetal bovine serum, 100 U/mL of penicillin and 100 ng/mL of streptomycin. Isolated MSCs were seeded in uncoated plastic dishes with same medium. The culture was maintained at 37 °C in a humidified atmosphere of 95% air and 5% CO_2_. After three to four days in culture, the non-adherent cells were removed, and the adherent cells were maintained in culture until they reached 80% confluence. The passage was performed using 0.5% trypsin–EDTA (#15400054, Gibco). Written informed consent was obtained from each patient. All procedures were approved by the Ethics Committee of the Kyoto University Graduate School of Medicine (G0325) and carried out in accordance with the Ethics Guidelines form Medical and Health Research Involvingt Human Subjects of the Ministry of Education, Culture, Sports, Science and Technology of Japan and the Ministry of Health, Labour and Walfare of Japan.

### Isolation of HUVECs

Separation and isolation of HUVECs were performed as previously described^22^. Briefly, the vein in the umbilical cord was flushed with PBS and one end of the vein was clamped. Collagenase was added into the other end of the vein until moderate distention of the vein was observed, and the cord was then incubated at 37 °C for 40 min. After incubation, the cord was massaged gently; the clamped end was then cut above the clamp and cells were collected into 50 mL tubes. Tubes were centrifuged at 1500 rpm for 5 min and the supernatant was aspirated. The pellet was resuspended in 6 ml of HuMedia-EG2 (#KE-2150S, Kurabo) and plated in 6 cm plates. The culture was maintained at 37 °C in a humidified atmosphere of 95% air and 5% CO_2_. The passage was performed using 0.5% trypsin–EDTA.

### Generation of three-dimensional placental bud in vitro

To generate the placental organ bud, we used the methods reported by Takebe et al.^23^. iPSCs were differentiated with BMP4 to a stage equivalent to day 4. 1.0 × 10^6^ human iPSC-derived trophoblast-like cells, 0.8 × 10^6^ to 1.0 × 10^6^ HUVECs and 2.0 × 10^5^ human MSCs were combined and centrifuged at 1500 rpm for 5 min at room temperature. The supernatant was discarded and the pellet was resuspended in 1 mL of a 1:1 mixture of HuMedia-EG2 (0.5 mL) and conditioning medium from HUVECs (0.5 mL). The final concentration of iPSC-derived trophoblast-like cells was 1.0 × 10^6^ cells/mL. 1 mL of mixed cells was then plated in each well of a pre-solidified Geltrex-coated 24-well plate (iPSCs: 1.0 × 10^6^ cells/well). Fluorescent images of organ buds from 409B2 iPSCs were captured by Olympus IX71 Inverted System Microscope.

### Quantitative real-time PCR

Quantitative RT-PCR was used to determine the relative levels of gene expression (LightCycler 480 System, Roche Life Sciences). Primer sequences used for amplifications are shown in Table 1. Unless otherwise stated, each experiment was performed in triplicate, and all results were normalized against *GAPDH*. Relative mRNA expression levels were determined according to the comparative cycle threshold (ΔΔCT) method. SYBR Green was used to detect amplification.

**Table 1:** Primer sequences used for qPCR.

	Forward	Reverse
*GAPDH*	5'-GAGTCAACGGATTTGGTCGTATTGG-3'	5'-GCCATGGGTGGAATCATATTGGAAC-3'
*SOX2*	5'-TAAGTACTGGCGAACCATCTCTGT-3'	5'-TTGGGATCGAACAAAAGCTATTATAA-3'
*NANOG*	5'-GCAGAAGGCCTCAGCACCCTA-3'	5'-AGGTTCCCAGTCGGGTTCA-3'
*POU5F1*	5'-GAGGAGTCCCAGGACATCAA-3'	5'-AACGGCAGATAGTCGTTTGG-3'
*CDX2*	5'-ATCACCATCCGGAGGAAAG-3'	5'-TGCGGTTCTGAAACCAGATT-3'
*PPARG*	5'-TGCCAAAAGCATTCCTGGTT-3'	5'-ATTCATCAAGGAGGCCAGCA-3'
*KRT7*	5'-CAGGCTGAGATCGACAACATC-3'	5'-CTTGGCACGAGCATCCTT-3'
*TP63*	5'-CCGCCGTCCAATTTTAATCA-3'	5'-CACAGATCCGGGCCTCAA-3'
*CGB3*	5'-CCCACAACCCCGAGGTATAAAG-3'	5'-ATGCTCAGCAGCAGCAACAG-3'
*ERVW1*	5'-CACATGGCCCAAGATTCCAT-3'	5'-AGATGGTGGCAAGCCTCGTA-3'
*HLAG*	5'-AGATCTCCAAGCGCAAGTGT-3'	5'-CCTTCCCGTTCTCCAGGTAT-3'

### Immunocytochemistry

Induced trophoblastic cells were grown in an eight-well chamber slide. Cells were fixed in 4% paraformaldehyde for 10 min. After being washed with PBS, slides were incubated with 10% normal goat serum (#50062Z, Life Technologies) for 30 min at room temperature. Thereafter, slides were incubated with primary antibodies overnight at 4 ˚C. The primary antibodies used and their concentrations were as follows: CK7 (#ab181598, abcam, 1:100 dilution), HLAG (#ab7758, abcam, 1:100 dilution). Slides were incubated with a secondary antibody (Alexa Fluor 488, abcam, 1:500 dilution) and then mounted with ProLong Gold Antifade Reagent with 40,6-diamidino-2-phenylindole (P36935, Molecular Probes). Images were captured by Olympus BX51 microscope and DP-2 BSW software system.

### Analysis of hCG

The media from induced trophoblast cells were collected for ELISA analysis and stored at − 20 °C. The secreted hCG was assayed using a Human hCG ELISA kit (abcam, #ab100533) according to the manufacturer’s instructions.

### Immunohistochemistry

Immunohistochemistry was performed using a Histofine Simple Statin MAX PO (M) (#41431F, Nichirei) or Histofine Simple Stain MAX PO (R) (#414141F, Nichirei) according to the manufacturer’s instructions. Paraffin-embedded specimens were cut into 4 μm-thick sections. Tissue sections were deparaffinized in xylene (3 × 3 min) and dehydrated through graded alcohols (100%, 100%, 95% and 95%) to water. Antigens were retrieved by the following methods. For CK7, HLAG and hCG staining, the samples were boiled in citrate buffer (pH 6.0) using microwaves for 15 min. For CD31 and CD90 staining, the samples were boiled in Tris–EDTA buffer (pH 9.0) using microwaves for 15 min. Sections were allowed to cool for 30 min at room temperature. To block endogenous peroxidase activity, sections were treated with 3% H2O2 in methanol for 15 min. For staining with the mouse primary antibodies (CK7, HLA-G, and CD31), the sections were then incubated with mouse anti-CK7 monoclonal Abs (#MA1-06,316, Thermo Fisher Scientific, 1:100 dilution), anti-HLAG Ab (#ab76869, abcam, 1:100 dilution), and mouse anti-CD31 antibody (#ab9498, abcam, 1:25 dilution) overnight at 4 °C. On the next day, the sections were incubated with Histofine Simple Statin MAX PO (M) for 30 min at room temperature. For staining with the rabbit primary antibodies (hCG and CD90), the sections were treated with 3% H_2_O_2_ in methanol, then incubated with Rabbit Anti-Human Chorionic Gonadotropin (#A0231, DAKO, 1:200 dilution) or rabbit anti-CD90 antibody (#ab133350, abcam, 1:100 dilution) overnight. On the next day, they were incubated for 30 min with Histofine Simple Stain MAX PO (R) at room temperature. Signals were generated by incubation with diaminobenzidine (DAB, #415171, Nichirei). Nuclei were counter-stained with hematoxylin (#30002, Muto Pure Chemicals). Images were captured using an Olympus BX51 microscope and the accompanying DP-2 BSW software package.

### Transplantation of placental organ buds into immunodeficient mice

Female NOD/ShiJic-scid Jcl (NOD/SCID) are immunodeficient mice lacking mature lymphocytes, functional macrophages, and circulating complements^24^; they are widely used in transplant experiments^25^. We purchased NOD/SCID mice from CLEA Japan for our transplant experiments. Animals were maintained under specific pathogen-free conditions. Pseudo-pregnant recipient mice were prepared as described previously with some modifications^26^. Six-week-old immunodeficient NOD/SCID mice were used in this experiment. Two mg of progesterone (#28921-64, Nacalai Tesque) dissolved in sesame oil (#25620-65, Nacalai Tesque) was subcutaneously injected every day starting four days before transplantation (Fig. 3A). A placental bud composed of 0.5 × 10^6^ human iPSC-derived trophoblast-like cells, 0.4–0.5 × 10^6^ HUVECs and 1.0 × 10^5^ human MSCs in Geltrex-coated 24-well plates were prepared for transplantation. On the day of transplantation, laparotomy was performed under general anesthesia (isoflurane, Pfizer), and the proximal side of the uterus was ligated with #5-0 silk to prevent dislocation of the transplantation. A placental bud was slowly injected into the uterine lumen using an 18 gauge needle. The puncture in the uterus was sutured with #5-0 nylon and the abdomen was closed with #5-0 silk. After the operation, 100 ng of estradiol-17β (#14514-74, Nacalai Tesque) dissolved in sesame oil was subcutaneously injected. Injection of progesterone was continued through the sixth day after transplantation. On the seventh day after transplantation, mice were sacrificed. Each uterus was collected and fixed in 4% paraformaldehyde overnight. All animal studies were approved by Kyoto University Animal Research Committee (Med Kyo 17118). Animal experiments were performed in accordance with the Regulations on Animal Experimentation at Kyoto University.

### Statistical analysis

Values are expressed as means ± SD. Data were analyzed by unpaired Student’s t-test unless indicated otherwise. *P*-values less than 0.05 were regarded as statistically significant.

## Supplementary Information

Supplementary Information.
