# Supplementary material for: Three-dimensional human placenta-like bud synthesized from induced pluripotent stem cells
Source: Sci Rep. 2021 Jul 8;11:14167. doi: 10.1038/s41598-021-93766-9 (PMC8266876; doi:10.1038/s41598-021-93766-9)
Supplement: Supplementary file 1 — Supplementary Information. [file 41598_2021_93766_MOESM1_ESM.pdf]

Three-dimensional human placenta-like bud synthesized from induced pluripotent stem cells

Mai Sato, Asako Inohaya, Eriko Yasuda, Haruta Mogami, Yoshitsugu Chigusa, Kaoru Kawasaki, Yosuke Kawamura, Yusuke Ueda, Hiroshi Takai, Masaki Mandai, and Eiji Kondoh*

Kyoto University Graduate School of Medicine, Department of Gynecology and Obstetrics

Kyoto, Japan

*** Corresponding author:** Eiji Kondoh, M.D., PhD.

Address: Kyoto University Graduate School of Medicine, Department of Gynecology and Obstetrics

54 Shogoin Kawahara-cho, Sakyo, Kyoto, 606-8507, Japan
Tel: 81-75-751-3269, Fax: 81-75-761-3967
Email: kondo@kuhp.kyoto-u.ac.jp

Funding sources: This work was supported by Grants-in-Aid for Scientific Research from the Ministry of Education, Science, Culture and Sports, Japan (No. 17H04339).

Keywords: induced-pluripotent cells, trophoblasts, placental organ buds, and transplantation

Supplementary Fig. 1

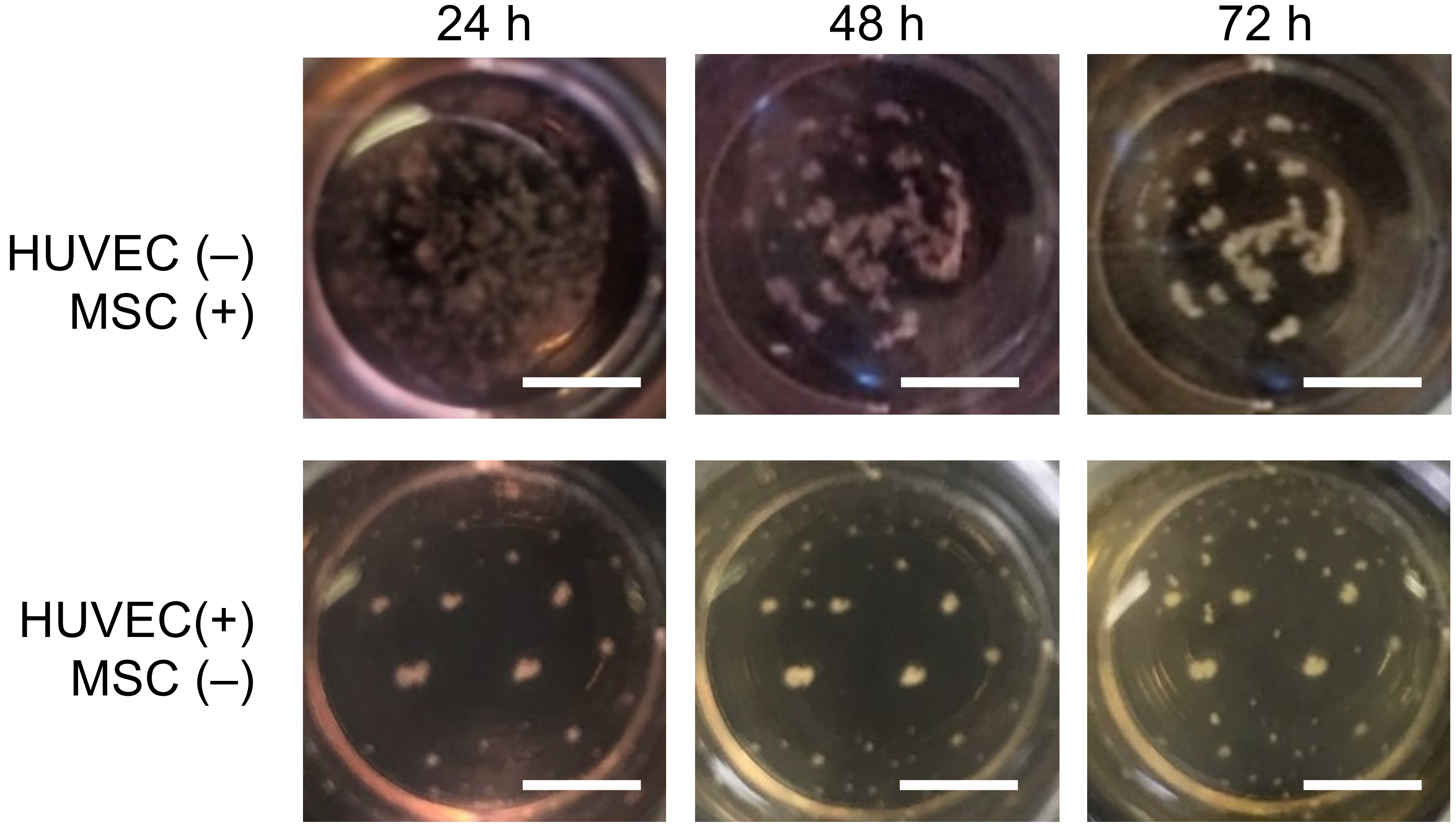

Failed organ buds. Representative images 201B7 iPSCs co-cultured with either HUVECs or MSCs for 24-72 h. iPSCs treated with BMP4 for 4 days (D4) were utilized. Bars, 5 mm.

Supplementary Fig. 2

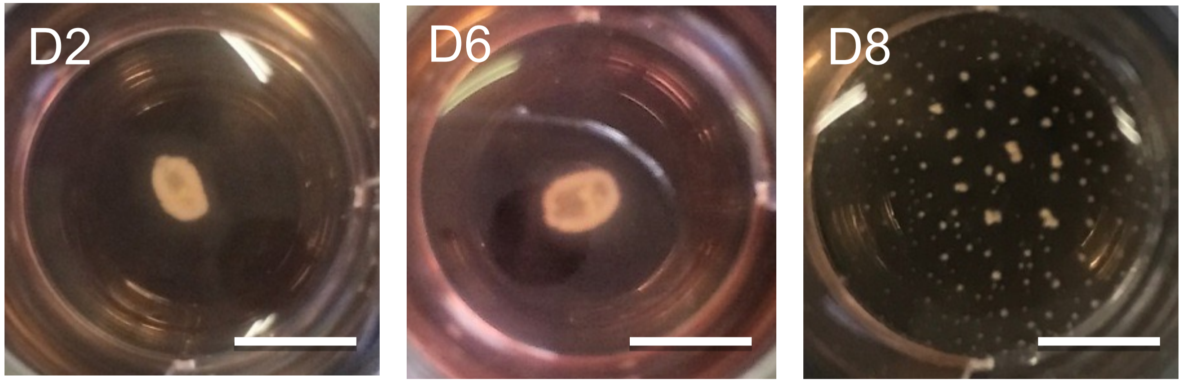

Images of placental organ buds from 201B7 iPSCs treated with BMP4 for 2 days (D2, left), 6 days (D6, middle) and 8 days (D8, right). Bars, 5 mm
